# Elimination of Cancer Stem-Like Cells and Potentiation of Temozolomide Sensitivity by Honokiol in Glioblastoma Multiforme Cells

**DOI:** 10.1371/journal.pone.0114830

**Published:** 2015-03-12

**Authors:** I-Chun Lai, Ping-Hsiao Shih, Chih-Jung Yao, Chi-Tai Yeh, Jacqueline Wang-Peng, Tai-Ngar Lui, Suang-En Chuang, Tsai-Shu Hu, Tung-Yuan Lai, Gi-Ming Lai

**Affiliations:** 1 Department of Oncology, Taipei Veterans General Hospital, Taipei, Taiwan; 2 Comprehensive Cancer Center, Taipei Medical University, Taipei, Taiwan; 3 Department of Pediatrics, Shuang Ho Hospital, Taipei Medical University, New Taipei City, Taiwan; 4 Cancer Center, Wan Fang Hospital, Taipei Medical University, Taipei, Taiwan; 5 Department of Internal Medicine, School of Medicine, College of Medicine, Taipei Medical University, Taipei, Taiwan; 6 Cancer Center, Shuang Ho Hospital, Taipei Medical University, Taipei, Taiwan; 7 Division of Neurosurgery, Wan Fang Hospital, Taipei Medical University, Taipei, Taiwan; 8 National Institute of Cancer Research, National Health Research Institutes, Miaoli, Taiwan; 9 Graduate Institute of Pharmacognosy, College of Pharmacy, Taipei Medical University, Taipei, Taiwan; Wake Forest University, School of Medicine, UNITED STATES

## Abstract

Glioblastoma multiforme (GBM) is the most common adult malignant glioma with poor prognosis due to the resistance to radiotherapy and chemotherapy, which might be critically involved in the repopulation of cancer stem cells (CSCs) after treatment. We had investigated the characteristics of cancer stem-like side population (SP) cells sorted from GBM cells, and studied the effect of Honokiol targeting on CSCs. GBM8401 SP cells possessed the stem cell markers, such as nestin, CD133 and Oct4, and the expressions of self-renewal related stemness genes, such as *SMO*, *Notch3* and *IHH (Indian Hedgehog)*. Honokiol inhibited the proliferation of both GBM8401 parental cells and SP cells in a dose-dependent manner, the IC_50_ were 5.3±0.72 and 11±1.1 μM, respectively. The proportions of SP in GBM8401 cells were diminished by Honokiol from 1.5±0.22% down to 0.3±0.02% and 0.2±0.01% at doses of 2.5 μM and 5 μM, respectively. The SP cells appeared to have higher expression of *O* ^6^-methylguanine-DNA methyltransferase (MGMT) and be more resistant to Temozolomide (TMZ). The resistance to TMZ could be only slightly reversed by MGMT inhibitor *O* ^6^-benzylguanine (*O* ^6^-BG), but markedly further enhanced by Honokiol addition. Such significant enhancement was accompanied with the higher induction of apoptosis, greater down-regulation of *Notch3* as well as its downstream *Hes1* expressions in SP cells. Our data indicate that Honokiol might have clinical benefits for the GBM patients who are refractory to TMZ treatment.

## Introduction

Malignant brain tumor is one of the most lethal cancers in adults. Based on WHO classification, grade I tumors are biologically “benign”, while grade II tumors are low-grade malignancies with longer clinical courses [1]. Grade III and IV are malignant gliomas that are lethal within few years and 9–12 months, respectively [2]. High-grade glioma (glioblastoma multiforme, GBM), the most frequent and malignant brain tumor in adults are typically resistant to radiotherapy and chemotherapy. The survival of GBM after surgery and radiotherapy was limited in one year. The chemotherapy, such as Temozolomide (TMZ), could further prolong the survival up to about twenty months [3], but most patients died within two years. Thus, searching for innovative therapeutic agents and developing novel strategies for refractory GBM patients are imperative and urgent.

TMZ is an alkylating chemotherapeutic agent that is currently used for the treatment of GBM. The therapeutic benefit of TMZ depends on its ability to alkylate/methylate nuclear acid, which most often occurs at the N-7 or O-6 positions of guanine residues [4]. This methylation damages the DNA replication by mispairing *O*-6-alkylguanine with thymine and triggers the death of tumor cells. It is demonstrated that cancer cells if express higher activity of *O* ^6^-methylguanine-DNA methyltransferase (MGMT), a DNA repairing enzyme to remove TMZ-DNA adduct, may diminish the therapeutic efficacy of TMZ and appear to be more resistant and refractory to TMZ therapy. Combined with MGMT inhibitor, O^6^-benzylguanine (*O* ^*6*^-BG), may enhance the cytotoxicity of TMZ [3]. However, the phase II clinical trial of the *O* ^6^-BG with TMZ did not show the response benefit for GBM except the patients with anaplastic astrocytoma (AA) [5]. The reasons for this discrepancy might be due to the different malignant behaviors including the presence of higher proportion of cancer stem cells (CSC) in GBM [6]. This clinical evidence gave rise to our motivation to search any therapeutics that is able to target on CSC of TMZ-resistant GBM.

The research for CSC or tumor-initiating cell (TIC) became an interesting field since 2000. No matter what kinds of the methods being used to identify the CSC, several solid tumor cell lines containing CSC had been reported in medulloblastoma, neuroblastoma, glioma, breast cancer, gastric cancer and colon cancer, etc. [7] It had also been demonstrated that the patients with recurrence and refractory to TMZ correlated with the higher proportion of CSC remained in tumor mass [6]. Therefore, searching for novel therapeutics targeting on the CSC becomes a new strategy to achieve a better clinical outcome of refractory GBM patients.

CSCs possess hallmarks of self-renewal capabilities, tumorigenesis and lineage differentiation into non-stem tumor cells as well as expression of multidrug-resistance proteins. In addition to using CD markers to identify CSCs, one common method to isolate the CSCs is the side population (SP) technique, which became more and more popular since 2004. This sorting technique to isolate SP cells by dual-wavelength flow cytometry is based on the capability of these cells to efflux the fluorescent DNA-binding dye Hoechst 33342 [8]. The phenotype of SP cells is characterized by high expression of breast cancer-resistant protein-1 (BCRP1 or ABCG2), one of ATP-binding cassette (ABC) transporters, which is associated with multidrug resistance in many cancers by pumping out the drugs [9]. Since multidrug resistance is an important characteristic of CSCs, it has also been shown that the sorted SP cells are enriched of CSCs [10]. Thus, sorting SP cells is postulated to be a rich source of CSCs and represents a technology platform for screening therapeutics that is able to target on CSCs.

Traditional Chinese medicines have been widely used in disease control for thousands of years. *Magnolia Officinalis*, also named Houpo in Chinese or Saiboku-tu in Japanese, is an ancient genus distributed mainly in South and East Asia as well as East and North America. Several active compounds derived from its barks had been identified, such as Magnolol, Honokiol, 4-*O*-methylhonokiol, Obovatol and other neolignan compounds, which possessed many diverse biological functions including improvement of allergic and asthmatic reactions [11]. Honokiol, one of the biphenolic bioactive components, possesses multifunctional activities, such as antioxidant, anti-inflammatory, chemopreventive and neuroprotective effects [12, 13, 14, 15]. As literature reported, Honokiol specifically inhibited PI3K/mTOR signaling activation in gliomas [16]. In addition, our recent findings suggested that Honokiol could travel through the blood-brain-barrier (BBB), indicating the potential for malignant glioma treatment [17]. However, there is little information about its anti-cancer effect against GBM cells, especially the effect on CSCs.

In this paper, we investigated the effects of Honokiol on the elimination of CSCs and the reversal of TMZ resistance in GBM8401 SP cells, and studied what underlying mechanisms are responsible.

## Material and Methods

### Cells and Materials

Honokiol (5,3′-Diallyl-2,4′-dihydroxybiphenyl), sulforhodamine B (SRB), Hoechst 33342, Verapamil, Reserpine, *O* ^*6*^-benzylguanine (*O* ^*6*^ *-*BG) and Temozolomide (TMZ) were purchased from Sigma Aldrich (Shanghai, China). Trizol RNA extraction reagent was obtained from MDBio Co. (Frederick, MD, USA). Human glioblastoma multiforme (GBM8401, BCRC No. 60163) and human glioblastoma/anaplastic astrocytoma (U-87 MG, BCRC No. 60360) cell lines were purchased from the Bioresource Collection and Research Center (BCRC) of Taiwan Food Industry Research and Development Institute. Other high grade reagents were purchased from commercial companies.

### Cell Culture

Human glioblastoma multiforme GBM8401 cells were maintained with Dulbecco's Modified Eagle Medium (DMEM) and human glioblastoma/anaplastic astrocytoma U87 MG cells were maintained in Eagle's Minimum Essential Medium (MEM). Cells were maintained in complete medium containing 10% fetal bovine serum (FBS), 1 X non-essential amino acids and 100 units/ml penicillin/streptomycin/_L_-glutamine (Gibco, Invitrogen Corporation, Grand Island, NY, USA) at 37°C in a humidified atmosphere.

### SRB Cell Viability Assay

Cytotoxicity effect of Honokiol was determined using a modified SRB colorimetric anticancer-drug screening assay [18]. Briefly, appropriate amounts of cells were seeded onto 96-well flat-bottomed plates in 200 μl of medium. After a 24 h incubation, the cells were treated with different concentrations of Honokiol (dissolved in DMSO, the final concentration was adjusted to less than 0.05%), followed by incubation for 3 more days at 37°C, 5% CO_2_. The medium was then aspirated, and cells were fixed onto the plastic substructure by the addition of 100 μl of 10% trichloroacetic acid (TCA) and incubation for 2 h at 4°C. The plates were washed five times with water to remove TCA and air-dried for at least 1 h. This was followed by staining with 200 μl of 0.065% SRB (sulforhodamine B) for 30 min at room temperature, washing five times with 1% acetic acid to remove unbound dye, and subsequently air-drying. Bound dye was dissolved with 200 μl of 10 mM Tris base (pH 10.5). Absorbance was read using a spectrophotometer at 570 nm.

### Isolation of the Side Population (SP) and Non-SP (NSP) Cells

Identification of side population was performed with slight modification as previously described [19]. GBM8401 or U-87 MG cells (10^6^/ml) was incubated in medium containing Hoechst 33342 (5 μg/ml) with or without positive control (ABC transporter inhibitor, Verapamil or Reserpine) for 3 h at 37°C. After incubation, the cells were analyzed using a Fluorescent-Activated Cell Sorting system with a DIVA software (FACSAria III, Becton Dickinson). Live cells were gated using propidium iodide and analyzed using a combination of a 450 nm (Hoechst Blue) and a 675 nm (Hoechst Red) filter after excitation with a 350 nm NUV laser. The SP cells were definitely identified upon the treatment of specific inhibitors against ATP-binding cassette transporters. The major cell population, named non-SP (NSP) cells, was significantly stained with Hoechst 33342. Both SP and non-SP cells were sorted and harvested respectively for further study. SP cells were maintained with HEscGro medium (Millipore, Billerica, MA, USA) containing epidermal growth factor (EGF, 10 ng/ml) plus basic fibroblast growth factor (bFGF, 8 ng/ml) but without any serum, and non-SP cells were incubated with DMEM complete medium.

### Flow Cytometric Analysis of Differentiation and Cell-Surface Markers

Single cell suspensions were selected using a 4-laser FACScalibur (BD Science) and stained with a variety of antibodies against CD markers that are highly expressed in cancer stem cells: anti-human CD24-FITC, anti-human CD26-FITC, anti-human CD29-FITC, anti-human CD90-FITC, anti-human CD133-PE, anti-human CD44-PE. Parental, SP and non-SP cells were incubated with specific antibodies mentioned above for 1 h, and then analyzed with flow cytometry after wash. In case of studying the differentiation capability of SP cells, the differentiated SP (d-SP) cells were obtained after culture in complete medium for one week. The d-SP, SP and parental cells were then incubated with fluorescent dye-conjugated antibodies such as anti-GFAP-PE and anti-MAP2B-Alexa Fluor488, respectively, for 1 h. The intensities of cellular fluorescence were then analyzed with flow cytometry after wash. All the fluorescent antibodies were purchased from BD Biosciences, San Jose, CA, USA, except the anti-human CD24-FITC (eBioscience, San Diego, CA, USA).

### Reverse Transcription PCR analysis

Cells were treated with Honokiol or Temozolomide for 48 h, the total cellular RNA were isolated using MasterPure RNA Purification Kit (Epicentre Biotechnologies, Madison, WI, USA) according to the manufacture’s protocols, and then quantified by an absorbance at 260 nm. RNA purity was determined using A260/A280 ratio (average ≥ 1.8). Total RNA from each specimen was first reverse-transcribed into cDNA using a MMLV Reverse Transcriptase 1st-Strand cDNA Synthesis Kit (Epicentre Biotechnologies), and then the target genes were amplified using Taq DNA Polymerase Master Mix (Ampliqon, Lars Quist, Denmark). Specimens were subjected to 30 cycles of amplification (cDNA synthesis at 37°C for 1 h followed by pre-denaturation at 94–C for 2 min, and the PCR amplification was performed with denaturation at 94 C for 30 sec, annealing at 58–60°C for 30 sec, extension at 72°C for 1 min, and the final extension at 72°C for 10 min). PCR products were mixed with EZ-Vision DNA Dye (Amresco, Solon, OH, USA) and then electrophoresed on a 1.8% agarose gel, finally visualized by UV-Visible Transilluminators System. The data were then quantified with Image J software.

### Western Blotting

After treatment, the cell lysates were prepared using ReadyPrep Protein Extraction Kit (Bio-Rad) according to instructions provided. Total cell lysates (20 μg) were separated electrophoretically by a 10% polyacrylamide SDS-PAGE gel and transferred onto a polyvinylidene fluoride membrane using the Bio-Rad Mini-Protean transfer system. The membrane was cropped and further incubated overnight at 4°C with specific antibodies against CD133 (St John's Laboratory Ltd, London, UK), MGMT (Cell Signaling Technology, Beverly, MA, USA) and tubulin (Abcam, Cambridge, MA, USA), respectively. After incubation with primary antibodies, the cropped membranes were washed with TBST 3 times and then incubated with horseradish peroxidase-labelled secondary antibody for 90 minutes at room temperature. After washing with TBST 3 times, final detection was performed with enhanced chemiluminescence (ECL) Western blotting reagents (Minipore) and BioSpectrum Imaging System (UVP, Upland, CA, USA).

### Cell Cycle Evaluation

Cells were treated with Honokiol or Temozolomide for 48 h. At the end of incubation, cells were harvested and fixed with 70% ethanol overnight and then incubated with a 25 μg/ml PI, 0.5% Triton X-100, and 0.2 μg/ml RNase A solution for 30 min after washing with cold PBS. Finally, the cells were resuspended in 500 μl PBS and then analyzed by flow cytometry (Cytomics FC500, Beckman Coulter). Data were collected from 10,000 cells and evaluated by CXP softwares.

### Statistical Analysis

All experiments were performed at least three times and the data are presented as the mean ± SD. Statistically significant differences were based on comparison with the untreated groups, and were calculated by a one- or two-way analysis of variance (ANOVA) and Student’s *t*-test statistical software. Values of p<0.05 were considered significant.

## Results

### Isolation of Side Population (SP) Cells from GBM8401 and U-87 MG Cell Lines

**Fig 1:**
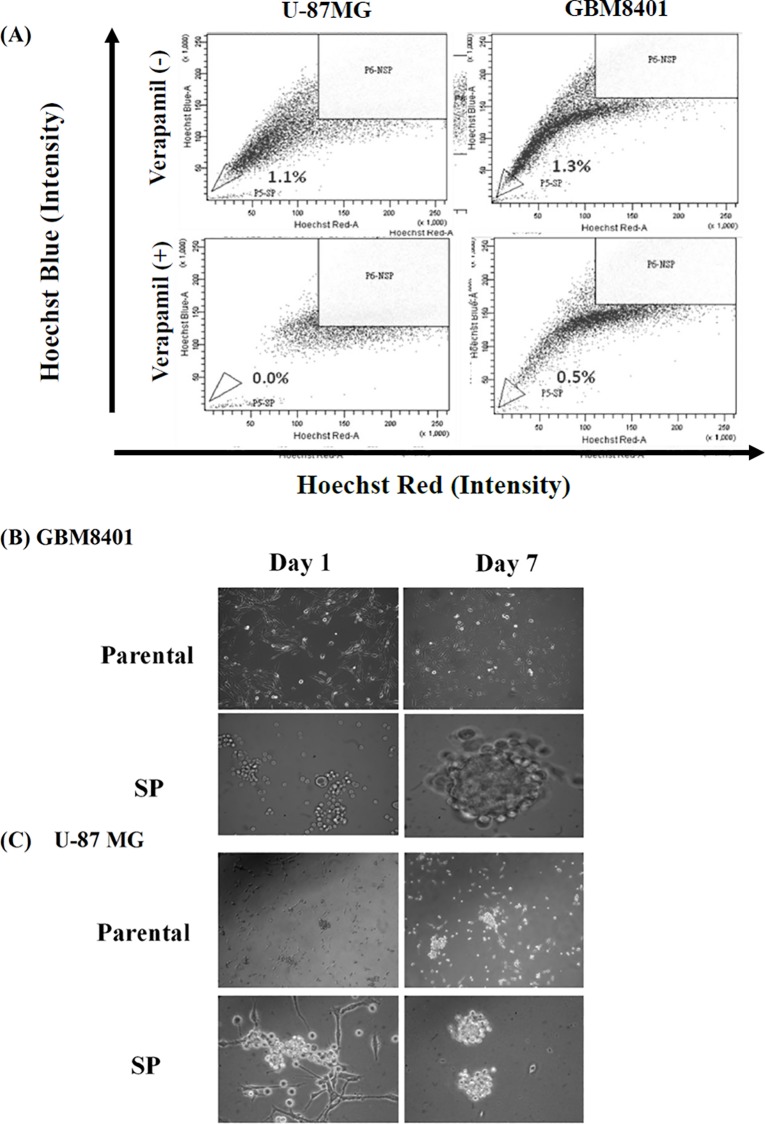
The Side Population (SP) analysis of GBM8401 and U-87 MG cancer cells. (A) The SP percentage was analyzed in GBM8401 and U-87 MG cells by Hoechst staining and flow cytometry. The cells were incubated with the absence or presence of specific inhibitor Verapamil (100 μM) for 3 h. SP assay was performed followed by staining the cells with Hoechst33342 (5 μg/ml) for 90 min. The morphology of GBM8401 (B) and U-87 MG (C) parental and SP cells were monitored and photographed after the performance of SP Flow cytometry. The SP cells were incubated in serum-free conditioned medium for 1 week and the spheroid morphology was observed. The SP cells were differentiated into non-SP (NSP) cells for long-term culture in complete serum containing medium.

The proportion of Verapamil-sensitive SP cells of GBM8401 or U-87 MG was analyzed by flow cytometry based on the exclusion of the DNA binding Hoechst 33342 dye. As shown in Fig. 1, there are 1.3±0.2% and 1.7±0.3% of SP cells detected in U-87 MG and GBM8401 cells, respectively. Those SP cells were then cultured in a suspension system with stem cell culture medium. The spheres appeared in the suspension were distinctly observed on day 7–10, which could be harvested for subsequent molecular assays.

### Differentiation Capability of SP Cells

**Fig 2:**
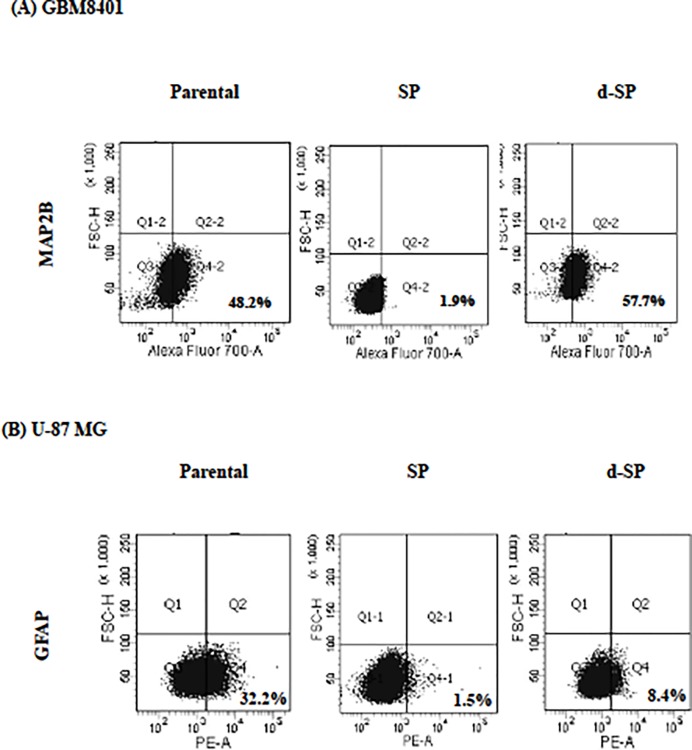
The differentiated SP cells show similar phenotype as that of parental cells. Parental, SP and differentiated SP (d-SP) cells were harvested to analyze the expression of specific marker protein such as microtubule-associated protein 2B (MAP2B) in GBM8401 (A) and glial fibrillary acidic protein (GFAP) in U-87 MG (B) cells. Signals were adjusted by subtracting the background of respective isotype control.

In case of studying the differentiation capability of SP cells, the differentiated SP (d-SP) cells were obtained after culture in complete medium for one week. As shown in Fig. 2(A), GBM8401 SP cells possessed much lower expression of neuronal marker MAP2B (2±0.4%), indicating their undifferentiated status. When the GBM8401 SP cells were differentiated to d-SP cells, the expression of neuronal marker MAP2B became as high as that of parental cells (50±1.9% in parental and 55±2.7% in d-SP cells). Similarly, the expression of astrocyte-specific marker GFAP (glial fibrillary acidic protein) in U-87 MG SP cells (1.7±0.2%) was much lower than that in the parental cells (30±1.8%). When these U-87 MG SP cells were differentiated to d-SP cells, the expression of GFAP was markedly increased to 8±1.1% (Fig. 2(B)).

### Characterization of GBM8401 SP Cells with Cancer Stem Cell Surface Markers

**Fig 3:**
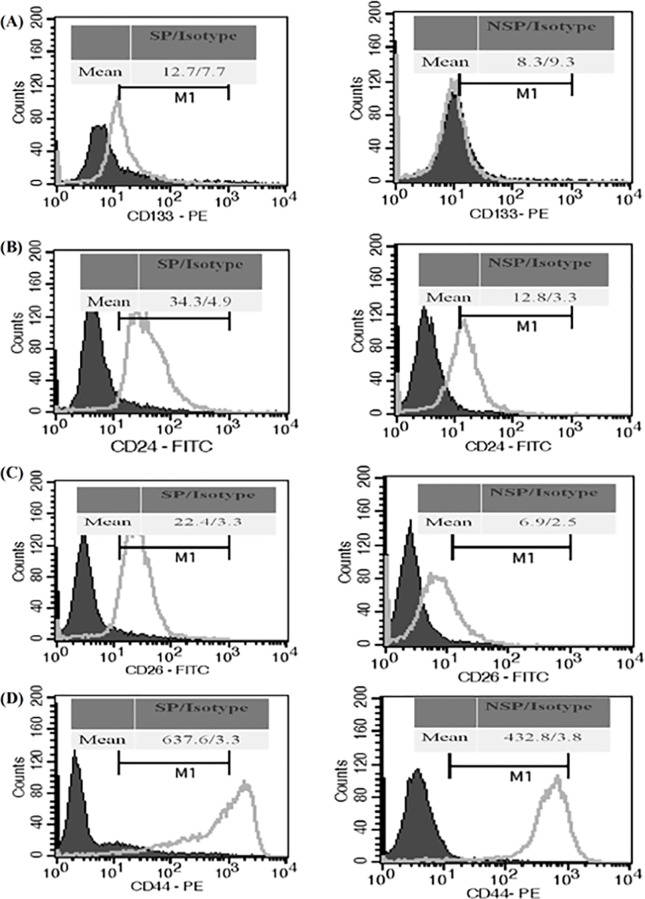
GBM8401 SP cells express higher level of stemness CD markers than the parental and non-SP (NSP) cells. GBM8401 SP and non-SP cells were sorted and then incubated with specific anti-CD marker antibodies or with isotype control for 1 h, respectively. After wash with PBS and centrifugation, cells were re-suspended with PBS and analyzed by flow cytometry. The histograms of flow cytometry analysis of CD133 (A), CD24 (B), CD26 (C), and CD44 (D) were shown. The expressions of the above stemness markers in SP cells were significantly higher than those in non-SP cells. However, the expressions of other markers, such as CD29, CD90, and SSEA-4, were not apparently different (data not shown).

CD24, CD26, CD29, CD44, CD90 and CD133 have been widely used to identify CSCs of solid tumors [20]. The expression of these CD markers in the fresh SP and non-SP cells sorted from GBM8401 cells were shown in Fig. 3. The expressions of CD24, CD26, CD44 and CD133 in the SP cells were significantly (p<0.05) higher than those in the non-SP cells, the degree of folds were 1.8±0.2, 2.5±0.3, 1.7±0.1 and 1.8±0.2, respectively. There were no significant differences in the expression of the other two markers, CD29 and CD90 (data not shown).

### The Expression of Stemness Gene Is Different in SP and non-SP (NSP) Cells

**Fig 4:**
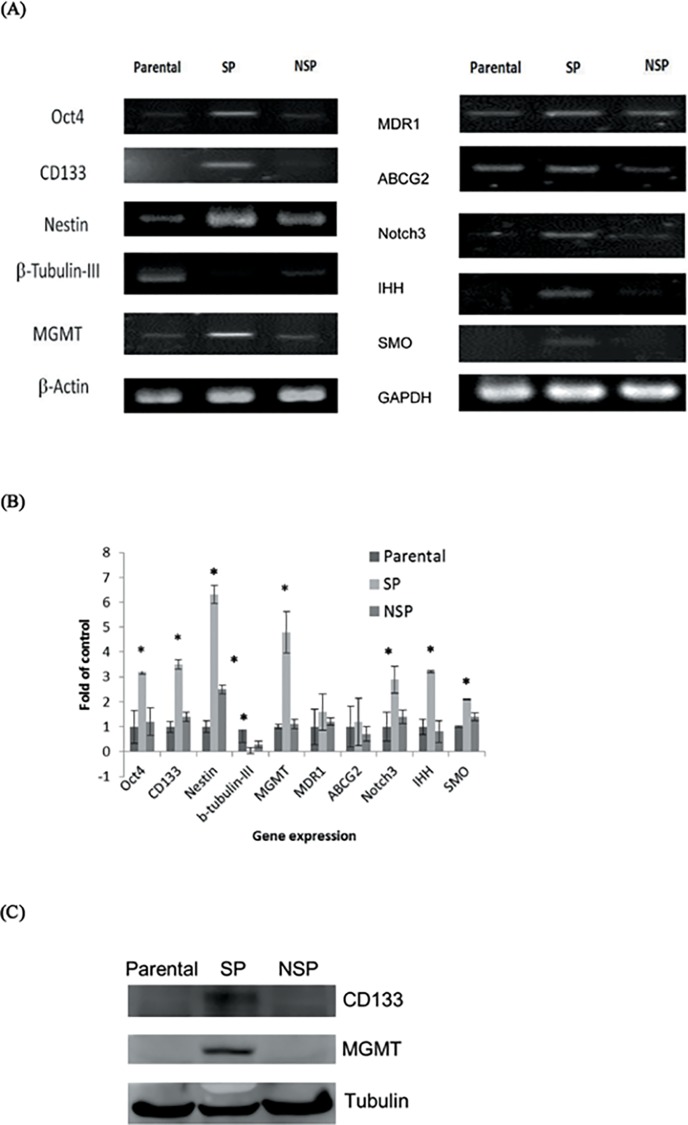
The expression of stemness genes in GBM8401 parental, SP and non-SP (NSP) cells. (A) RT-PCR analysis for the expressions of stemness markers (*CD113*, *Oct-4*), neuronal differentiation markers (*nestin*, *β-tubulin III*), DNA-repair enzyme (*MGMT*), stemness signaling pathway (*Notch3*, *IHH*, *SMO*) and drug-resistance genes (*ABCG2*, *MDR1*, *MGMT*). Except the neuronal differentiation marker *β-tubulin III*, all of the stemness genes expressions in SP cells were significantly higher than those in parental and non-SP (NSP) cells. The *β-Actin* and *GAPDH* were used as loading control. (B) Quantitative and statistical analysis data of (A) and two independent similar experiments. *, p<0.05 as compared with parental cells. (C) Western blot analysis for the protein levels of GBM CSC marker CD133 and TMZ resistance-associated protein MGMT. Both CD133 and MGMT protein levels were obviously much higher in the SP cells compared with the parental and non-SP cells. The Tubulin was used as loading control.

To further investigate the differential expression of stemness genes in SP and non-SP cells, we examined the mRNA expression of *Oct-4*, *CD133*, *Nestin*, *β-tubulin-III*, *Notch3*, *IHH* and *SMO* in GBM8401 parental, SP and non-SP cells. Except the *β-tubulin-III*, the expressions of mRNAs mentioned above were all significantly (p<0.05) higher in the SP cells as compared with the parental and the non-SP cells (Fig. 4(A) and 4(B)). Furthermore, the mRNA level of the multidrug resistant protein, such as *MDR1* and *ABCG2*, and the *MGMT* expression in the SP cells were also higher than those in the parental and non-SP cells (Fig. 4(A) and 4(B)). Consistent with the mRNA data, the protein levels of GBM CSC marker CD133 and TMZ resistance-associated protein MGMT were obviously much higher in the SP cells compared with the parental and non-SP cells (Fig. 4(C)).

### Honokiol Eliminates SP and Inhibits the Proliferation of GBM8401 Cells

**Fig 5:**
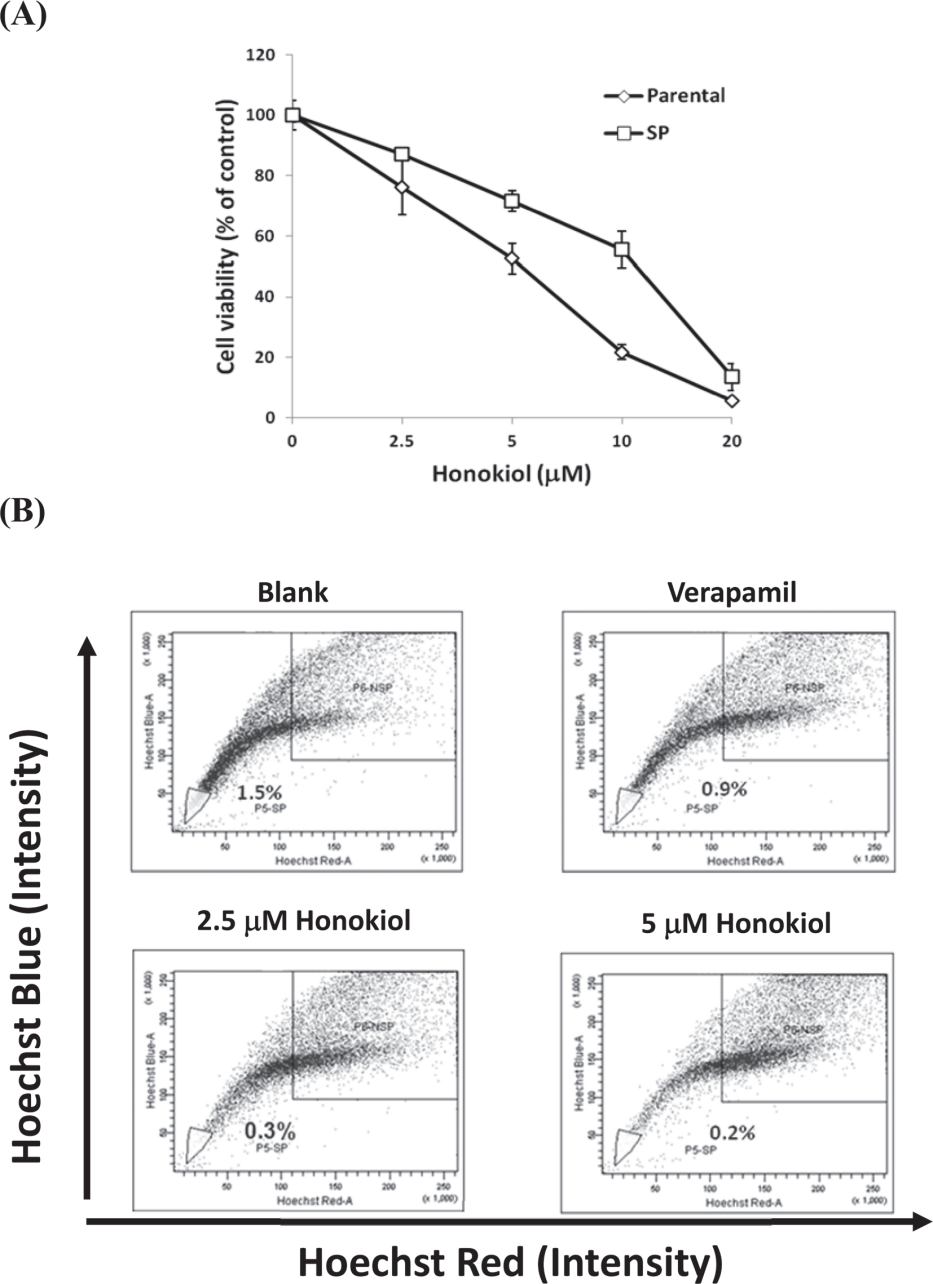
The effects of Honokiol on the cell viability and proportion of GBM8401 SP cells. (A) GBM8401 parental or SP cells were incubated with series concentrations of Honokiol for 48 h and the cell viability was examined by SRB assay. (B) GBM8401 cells were incubated with the absence or presence of specific inhibitor Verapamil (50 μM) and Honokiol (2.5 and 5 μM) for 48 h, respectively. After washed with PBS and centrifugation, SP assay was performed followed by staining the cells with Hoechst33342 (5 μg/ml) for 90 min.

The GBM8401 parental and SP cells were treated with different doses of Honokiol. The cell viability was determined by SRB assay and the proportion of SP cells was analyzed by flow cytometry. The result showed that Honokiol inhibited the proliferation of GBM8401 parental and SP cells in a dose-dependent manner (Fig. 5A). The GBM8401 SP cells were more resistant to Honokiol as compared with parental cells, the IC_50_ were 11.2±1.1 and 5.3±0.7 μM, respectively. The sensitivity of non-SP (NSP) cells to Honokiol was similar to that of parental cells (S1 Fig.). On the other hand, the proportion of the SP population in parental cells was diminished by Honokiol from 1.5±0.23% down to 0.3±0.02% and 0.2±0.02% at doses of 2.5 μM and 5 μM, respectively, as shown in Fig. 5B.

### Honokiol Further Enhances the Cytotoxicity and Apoptosis Induced by the Combination of Temozolomide and MGMT Inhibitor *O* ^*6*^-BG in GBM8401 SP Cells

**Fig 6:**
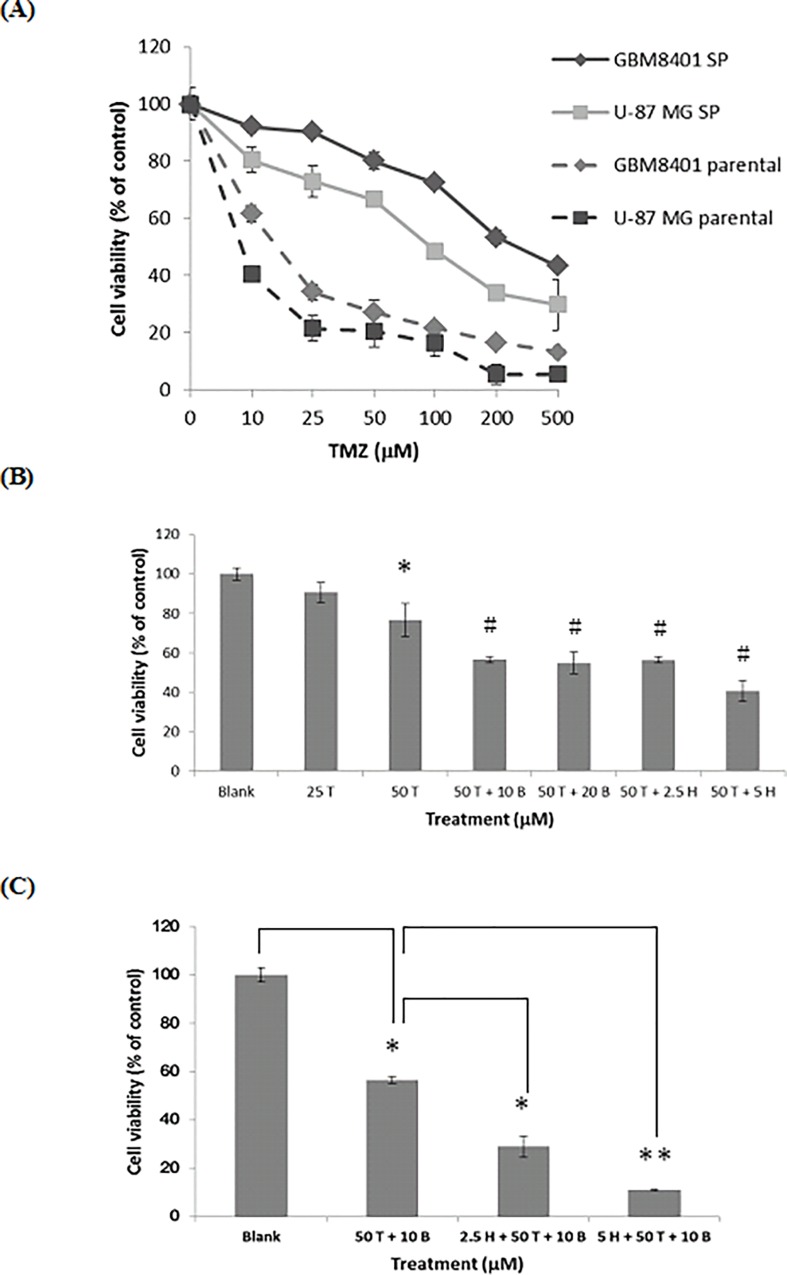
Effects of Honokiol in combination with TMZ plus *O* ^*6*^-BG on the cell viability of GBM8401 SP cells. (A) U-87 MG and GBM8401 parental cells and SP cells were treated with various concentrations of TMZ. Both GBM8401 and U87 MG SP cells were more resistant to TMZ than their parental cells. (B) The effects of *O* ^*6*^-BG (μM) or Honokiol (μM) on TMZ (μM)-suppressed GBM8401 SP cell viability. *, p<0.05 as compared with blank; #, p<0.05 as compared with 50 μM TMZ-treated group. No significant difference between each two pound sign groups. (C) The combination effects of Honokiol and TMZ plus *O* ^*6*^-BG on the cell viability of GBM8401 cells. *, p<0.05; **, p<0.01 as compared with other group indicated in the bar graph. The cell viability was determined by SRB assay with triplicate samples. H, Honokiol; T, TMZ; B, *O* ^*6*^-BG.

**Fig 7:**
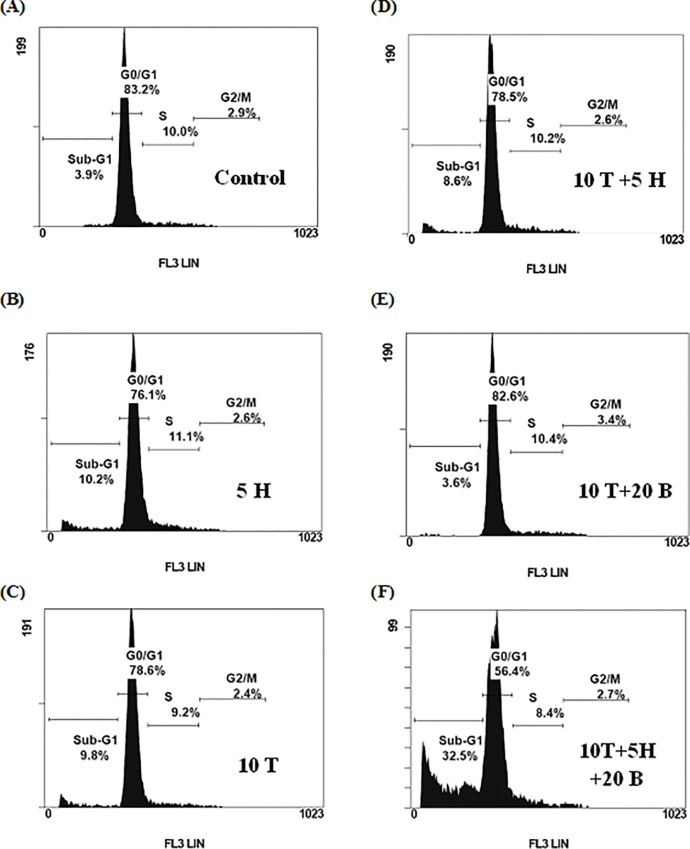
Apoptosis-enhancing effect of Honokiol on Temozolomide plus *O* ^*6*^-BG-treated GBM8401 SP cells. GBM8401 SP cells were treated with 0.05% DMSO (A), 5 μM Honokiol (B), 10 μM TMZ (C), 5 μM Honokiol combined with 10 μM TMZ (D), 10 μM TMZ plus 10 μM *O* ^*6*^-BG (E), and 5 μM Honokiol combined with 10 μM TMZ plus 10 μM *O* ^*6*^-BG (F) for 72 h, respectively. After harvest, cells were fixed and stained with PI for 30 min and the cell cycle was examined by flow cytometry. Honokiol enhanced *O* ^*6*^-BG combined with TMZ-mediated cytotoxicity through promoting the apoptotic cell death. H, Honokiol; T, TMZ; B, *O* ^*6*^-BG.

As Fig. 6A shows, GBM8401 SP cells were extremely higher resistant to TMZ than the parental cells, the IC_50_ was 490±5.3 μM and 15±0.4 μM, respectively. Similar result was also observed in the U-87 MG parental and SP cells. As the GBM8401 cells were relatively resistant to TMZ than U-87 MG cells, we then chose the GBM8401 cells to investigate the effects of Honokiol on this resistance of SP cells to TMZ. At the dose of 50 μM, TMZ only slightly decreased about 20% of the SP cell viability. Co-treatment with either *O* ^*6*^ *-*BG (10 μM) or Honokiol (5 μM), the cell viability of 50 μM TMZ-treated group was slightly decreased from about 80% to 60% and 40%, respectively (Fig. 6B). The co-treatment effect of 50 μM TMZ plus 10 μM *O* ^*6*^-BG could not be further enhanced by increasing the dose of *O* ^*6*^-BG to 20 μM (Fig. 6B). However, it could be significantly enhanced by Honokiol in a dose-dependent manner. The cell viability was further significantly decreased to 23% (p<0.05) and 10% (p<0.01) by Honokiol at dosages of 2.5 μM and 5 μM, respectively, as compared to 50 μM TMZ plus 10 μM *O* ^*6*^-BG-treated group (Fig. 6C). In line with this, only minimal apoptotic effects induced by single agent alone were observed and no substantial increase of sub-G1 percentages were observed if combined TMZ with either *O* ^*6*^ *-*BG or Honokiol. However, striking increases of apoptosis (about 30% sub-G1 cells, p<0.05) were found while cells were treated with TMZ (10 μM), *O* ^*6*^ *-*BG (10 μM) and Honokiol (5 μM) together (Fig. 7). The sub-G1 proportions of GBM8401 SP cells treated with Honokiol and TMZ with or without *O* ^*6*^ *-*BG were shown in Table 1.

**Table 1:** Sub-G1 proportion of GBM SP cell upon various treatments.

Treatment (μM)	sub-G1 (% of total cells)
Control	4±1.2
2.5 Honokiol	8±3.3
5 Honokiol	11±5.2
10 TMZ	9±1.9
10 TMZ + 2.5 Honokiol	10±2.2
10 TMZ + 5 Honokiol	11±3.9
10 TMZ + 10 *O* ^*6*^-BG	8 ±1.9
10 TMZ + 20 *O* ^*6*^-BG	10±2.7
10 TMZ + 2.5 Honokiol + 20 *O* ^*6*^-BG	27±2.1*
10 TMZ + 5 Honokiol + 20 *O* ^*6*^-BG	33±1.8*

*p<0.05 as compared with other treatments.

### Honokiol in Combination with *O* ^*6*^ *-*BG Further Inhibits Temozolomide-induced Notch3/Hes1 Cascade in GBM8401 SP Cells

**Fig 8:**
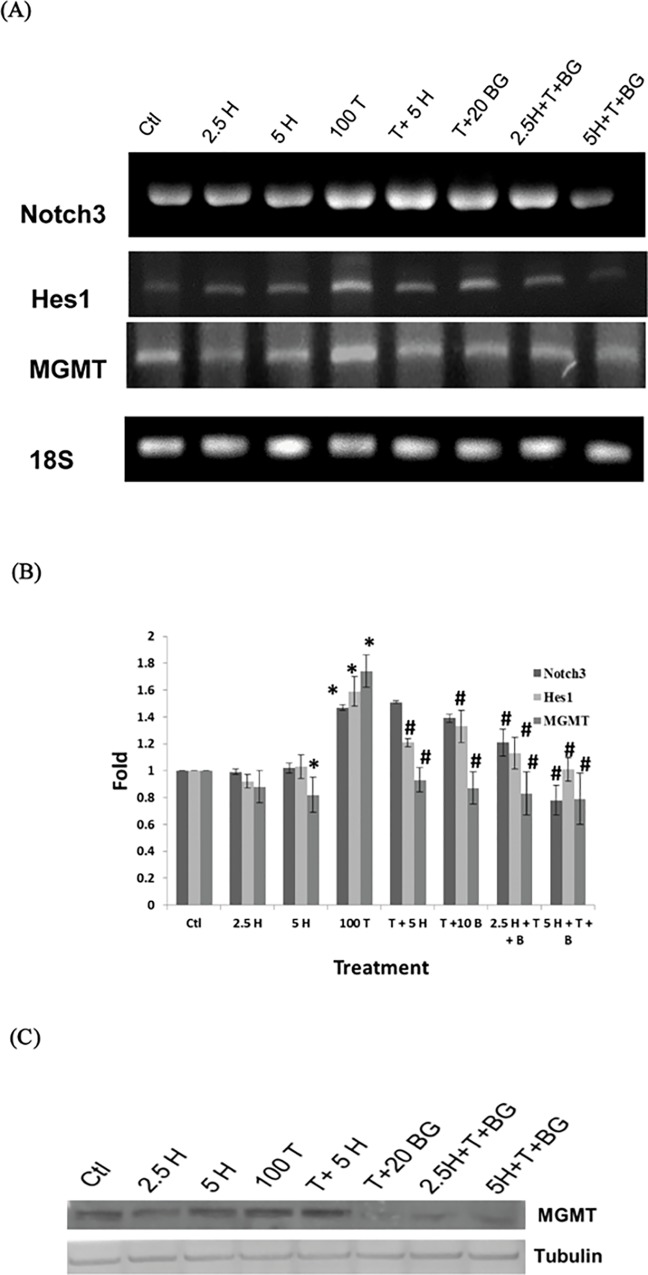
Honokiol in combination with *O* ^*6*^ *-*BG further inhibits Temozolomide-induced Notch3/Hes1 cascade in SP Cells. (A) RT-PCR analysis for the expression of *Notch3*, *Hes1* and *MGMT* mRNAs in SP cells. GBM8401 SP cells were treated with 2.5 and 5 μM Honokiol (lane 2 and 3, respectively), 100 μM TMZ (lane 4), 100 μM TMZ plus 5 μM Honokiol (lane 5), 100 μM TMZ plus 20 μM *O* ^*6*^-BG (lane 6), and 2.5 and 5 μM Honokiol combined with 100 μM TMZ plus 20 μM *O* ^*6*^-BG (lane 7 and 8, respectively) for 48 h. The *18S* was used as loading control. Honokiol in Combination with *O* ^*6*^ *-*BG Further suppressed Temozolomide-induced elevation of *Notch3* and *Hes1* mRNAs. (B) Quantitative and statistical analysis data of (A) and two independent similar experiments. (C) Western blot analysis for the protein level of MGMT in GBM8401 SP cells treated with agents as described in (A). The Tubulin was used as loading control. Ctl, DMSO mock control; H, Honokiol; T, TMZ; B, *O* ^*6*^-BG. *, p<0.05 compared with DMSO mock control group. #, p<0.05 compared with 100 μM TMZ-treated group.

As shown in Fig. 8(A) and 8 (B), the mRNA levels of *MGMT* as well as *Notch3* and its downstream *Hes1* were significantly (p<0.05) increased by TMZ (100 μM) treatment as compared with control group. Honokiol (5 μM) effectively abolished the TMZ-induced expressions of *Hes1* and *MGMT*. In the presence of *O* ^*6*^ *-*BG (20 μM), the protein level of MGMT was markedly diminished (Fig. 8 (C)). When combined with *O* ^*6*^ *-*BG (20 μM), Honokiol further (p<0.05) diminished the TMZ-induced expression of *Notch3* and *Hes1* mRNAs, indicating the potential role of Honokiol in combination with *O* ^*6*^ *-*BG for reversing the TMZ resistance in GBM SP cells.

## Discussion

The “side population” (SP) technique is a well-established method to isolate cancer stem–like cells. Recently, Fukaya and his colleagues had published an article using the SP technique to isolate cancer stem-like cells from various human glioblastoma cell lines [21]. Their study showed that SP cells had higher drug efflux capacity, more stemness genes expression and greater tumorigenesity as compared with non-SP cells. Similarly in this study, the SP isolated from GBM8401 cells appeared to have CSC properties. In addition to *Nestin*, *Notch-1* and *ABCG2*, we had also showed other stemness genes, such as *CD133*, *Oct4*, *MGMT* and *MDR1*, were more expressed in SP cells as compared with non-SP cells. Furthermore, SP cells could differentiate into different phenotype of cells with differentiation markers such as GFAP and MAP2B. The obviously much higher CD133 and MGMT protein levels of SP cells further characterized their stem-like cell properties, and therefore the GBM8401 SP represented an enriched CSC population.

Glioma CSCs could be isolated and identified by cell surface markers, such as Nestin and CD133, which were also highly expressed in our SP cells. CD133 is the most common cell surface markers for brain tumor stem cell identification and isolation; however, the percentages of CD133 positive cells isolated from human brain tumor tissues were not consistent, ranged from 3% to 45% [22]. Therefore, it is not convincible to characterize cancer stem cells by using only one specific cell surface marker [23]. Based on our findings, other markers such as CD24, CD26 and CD44 were also more expressed in GBM8401 SP cells compared to non-SP cells, ranged from 1.7 to 2.5 folds. The CSC properties of SP cells were further confirmed by the evidence of higher expressions of multiple stemness markers described above.

It was demonstrated that CD133-highly expressed cells located at the central part of tumor showed more resistant to TMZ in contrast to those located at the peripheral layer [6]. Accordingly, the TMZ resistant GBM had been shown to contain higher proportion of CD133^+^ cells and the TMZ resistant SP cells in this study also possessed higher level of CD133. Moreover, the SP cells possessed much higher level of MGMT, which correlated with the resistance to TMZ. Therefore, the resistance of GBM cells to TMZ might be due to the following two factors: (1) having more proportion of GBM stem cells, and (2) containing higher level of MGMT.

Honokiol is a biphenolic bioactive compound isolated from the genus *Magnolia Officinalis* and shows multifunctional antitumor activities, such as cell cycle regulation, induction of apoptosis and inhibition of metastasis in several cancers [24, 25, 26]. Herein, this is the first study to investigate the novel efficacy of Honokiol targeting on GBM stem-like cells. As expected, Honokiol significantly diminished the proportion of SP in GBM8401 cells in a dose-dependent manner. A phase II trial had shown that the MGMT inhibitor O6-BG seemed to exhibit no significant improvement on the TMZ sensitivity in patients with TMZ-resistant GBM [5]. In agreement with this, the resistance of GBM8401 SP cells to TMZ could not be substantially reversed by *O* ^*6*^-BG, although the MGMT protein level of SP cells in each *O* ^*6*^-BG-treeated group was markedly diminished. However, Honokiol significantly enhanced the cytotoxic effects of TMZ plus *O* ^*6*^-BG against SP cells, which were evidenced by marked induction of apoptosis. In the presence of Honokiol and *O* ^*6*^-BG, the TMZ-induced activation of *Notch3* and its downstream *Hes1* mRNAs were markedly suppressed. It has been suggested that the Notch signaling pathway plays an important role in proliferation, stem cell maintainance, and tumorigenesis [27]. As inhibition of Notch pathway had been shown to enhance sensitivity of CD133^+^ glioma stem cells to Temozolomide therapy [28]. Furthermore, inhibition of Hes1 also had been reported to enhance apoptosis of U-87 MG glioblastoma cells [29]. In our data, the TMZ-elevated Hes1 mRNA was only obviously reduced in the presence of Honokiol. Therefore, we deduced that the increased apoptosis might be due to the inhibition of Notch3/Hes1 pathway by Honokiol. This marked repression of Notch3 and Hes1 might be responsible for the enhanced effects of TMZ plus *O* ^*6*^-BG against GBM8401 SP cells by Honokiol. In accordance with our findings, Ponnurangam et al. recently reported Honokiol in combination with radiation could target CSC by inhibiting the Notch signaling pathway in colon cancer [30]. Therefore, it indeed shows the potential of using Honokiol in combination with chemo- or radio-therapy for cancer treatment including GBM.

Simultaneous inhibitions of MGMT gene expression as well as the Notch signaling pathway are strategically important for reversal of TMZ resistance in GBM CSCs. The present study indicated that Honokiol could play a very important role for the circumvention or reversal of the resistant events induced by TMZ treatment. These findings justify the future clinical trials of using Honokiol in combination with *O* ^*6*^ *-*BG for not only potentiation of TMZ efficacy but also elimination of TMZ-resistant CSCs. Thereby, the clinical outcome of TMZ refractory GBM patients may be substantially improved.

## Supporting Information

S1 Fig The effects of Honokiol on the cell viability of GBM8401 parental and NSP (non-SP) cells. GBM8401 parental or NSP cells were incubated with series concentrations of Honokiol for 48 h and the cell viability was examined by SRB assay.
(TIF)
