# Supplementary figures and images for: Elimination of Cancer Stem-Like Cells and Potentiation of Temozolomide Sensitivity by Honokiol in Glioblastoma Multiforme Cells
Source: PLoS One. 2015 Mar 12;10(3):e0114830. doi: 10.1371/journal.pone.0114830 (PMC4357432; doi:10.1371/journal.pone.0114830)

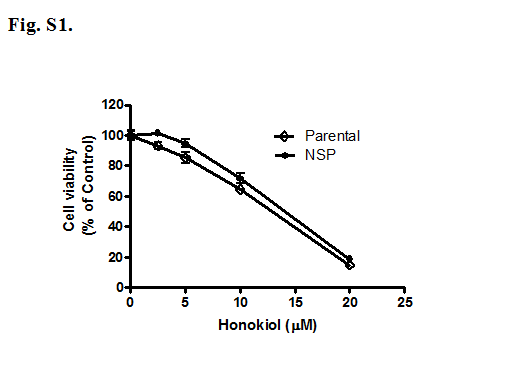

Supplement: S1 Fig — GBM8401 parental or NSP cells were incubated with series concentrations of Honokiol for 48 h and the cell viability was examined by SRB assay. (TIF) [file pone.0114830.s001.tif]
